# Injective mesenchymal stem cell-based treatments for knee osteoarthritis: from mechanisms of action to current clinical evidences

**DOI:** 10.1007/s00167-018-5118-9

**Published:** 2018-08-29

**Authors:** Silvia Lopa, Alessandra Colombini, Matteo Moretti, Laura de Girolamo

**Affiliations:** 1 Cell and Tissue Engineering Laboratory, IRCCS Istituto Ortopedico Galeazzi, Via R. Galeazzi 4, 20161 Milan, Italy; 2 Orthopaedic Biotechnology Lab, IRCCS Istituto Ortopedico Galeazzi, Via R. Galeazzi 4, 20161 Milan, Italy; 3 Regenerative Medicine Technologies Lab, Ente Ospedaliero Cantonale, Via Tesserete 46, 6900 Lugano, Switzerland; 4 Swiss Institute for Regenerative Medicine, Lugano, Switzerland

**Keywords:** Osteoarthritis, Inflammation, Mesenchymal stem cells, Intra-articular injection, Articular cartilage, Bone marrow concentrate, Stromal vascular fraction, Adipose tissue

## Abstract

**Purpose:**

Osteoarthritis (OA) represents a relevant social and economic burden worldwide. “Mesenchymal stem cells” or, as recently proposed, “medicinal signaling cells” (MSCs) have been recently introduced as injective treatments for OA with the aim of restoring joint homeostasis. The aim of this review is to provide the reader with the tools necessary to interpret the currently available clinical data, focusing on the MSC mechanisms of action which might help to clarify what we should expect from this treatment.

**Methods:**

Clinical studies reporting MSC injections for the treatment of knee OA, either freshly isolated or culture-expanded cells, have been included and commented in relation to the supposed therapeutic effect that MSCs might exert giving their supposed mode of actions.

**Results:**

The majority of the studies reports significant improvements in terms of pain and knee function compared to baseline values, up to 24 months of follow-up. Although these data support the expected therapeutic effect of this therapy giving the features of these cells, only 14% of the studies present a control group and more than one-third of them report the results on less than ten patients.

**Conclusions:**

Despite the constant presence of positive and satisfactory results in the studies analyzed, the complexity of MSC metabolism and related therapeutic effects as well as the weakness of most of the studies do not allow withdrawing definitive conclusions about the superiority of one tissue source over another, as well as about the best cell dose and the long-term durability of the effects of these procedures. Given the high potential value of these therapies in the treatment of OA, further studies accurately designed, carefully defining the type of patients to be included and pursuing minimal standard requirements in terms of follow-up, number of patients, and types of measurements should be conducted to finally assess the efficacy of MSC-based injective treatments.

## Introduction

Osteoarthritis (OA), the most widespread type of arthritis, is expected to become the fourth cause of disability by 2020 [1, 2], resulting in a relevant socioeconomic burden and affecting the gross domestic product of developed countries [3]. The establishment of adequate therapies able to counteract the progression of the disease and, hence, to prevent the loss of articular function and joint replacement is needed. In particular, the current conservative options, which include exercise and physiotherapy, and weight loss, with the use of analgesics and nutraceuticals, should be combined to yield more effective treatments. As symptoms escalate, anti-inflammatory drugs and intra-articular steroids can also be used to get pain relief and improve joint function [4]. However, in patients who do not respond to optimal conservative management, joint replacement represents the unique available therapeutic option. In this scenario, the development of efficacious conservative approaches would be particularly relevant to treat young individuals with early OA, since their more active and physically demanding lifestyle negatively correlates with the prosthetic implant survival [5].

### Overview on injective mesenchymal stem cell-based treatments

Treatments involving the use of mesenchymal stem cells (MSCs), harvestable with minimally invasive procedures particularly from bone marrow and adipose tissue, are on the rise for the conservative treatment of OA [6]. MSCs have been demonstrated to be safe [7] and, in case of failure, they do not preclude any additional future treatment. Given the presence of diffuse chondral damages in osteoarthritic joints, the most common way to deliver MSCs in these patients is intra-articular injection. They have been used both in one-step procedure, as non-expanded cells, after in vitro expansion. The in vitro step allows for the selection of a more homogeneous cell population, meeting the standard criteria for MSC identification [8]. Furthermore, the number of cells administered to the patient can be precisely determined, ensuring a high reproducibility of the clinical procedure. On the other hand, therapies based on expanded cells involve a higher cost of the treatment. Additional concerns are related to the extensive in vitro cell manipulation, resulting in their classification as an advanced-therapy medicinal product (ATMP) and in the subsequent need to satisfy rigorous regulatory requirements for clinical use [9, 10]. To overcome some of these limitations, it is possible to process by commercially available disposable devices both bone marrow and adipose tissue obtaining bone marrow aspirate concentrate (BMAC) and stromal vascular fraction (SVF) or micro/nano-fragmented adipose, respectively. These products do not imply substantial cell manipulation and, thus, are not considered ATMPs. This makes their use easier not only from the technical point of view, since they are obtained in a single stage at the point of care, but also given the less complex regulatory pathway that has to be followed, although a clear position of the regulatory agents concerning the application of BMAC and SVF intra-articularly is still missing. However, the amount of MSCs present in these concentrates is usually lower compared to the doses of expanded MSCs that are administered to the patients, although this does not necessarily imply an inferior efficacy of the treatment [11]. Indeed, progenitor cell concentrates are a mixed cell population, including erythrocytes, leukocytes, and endothelial cells, and this allows maintaining MSCs in contact with their physiological cell niche, which is supposed to enhance their performances.

**Fig. 1:**
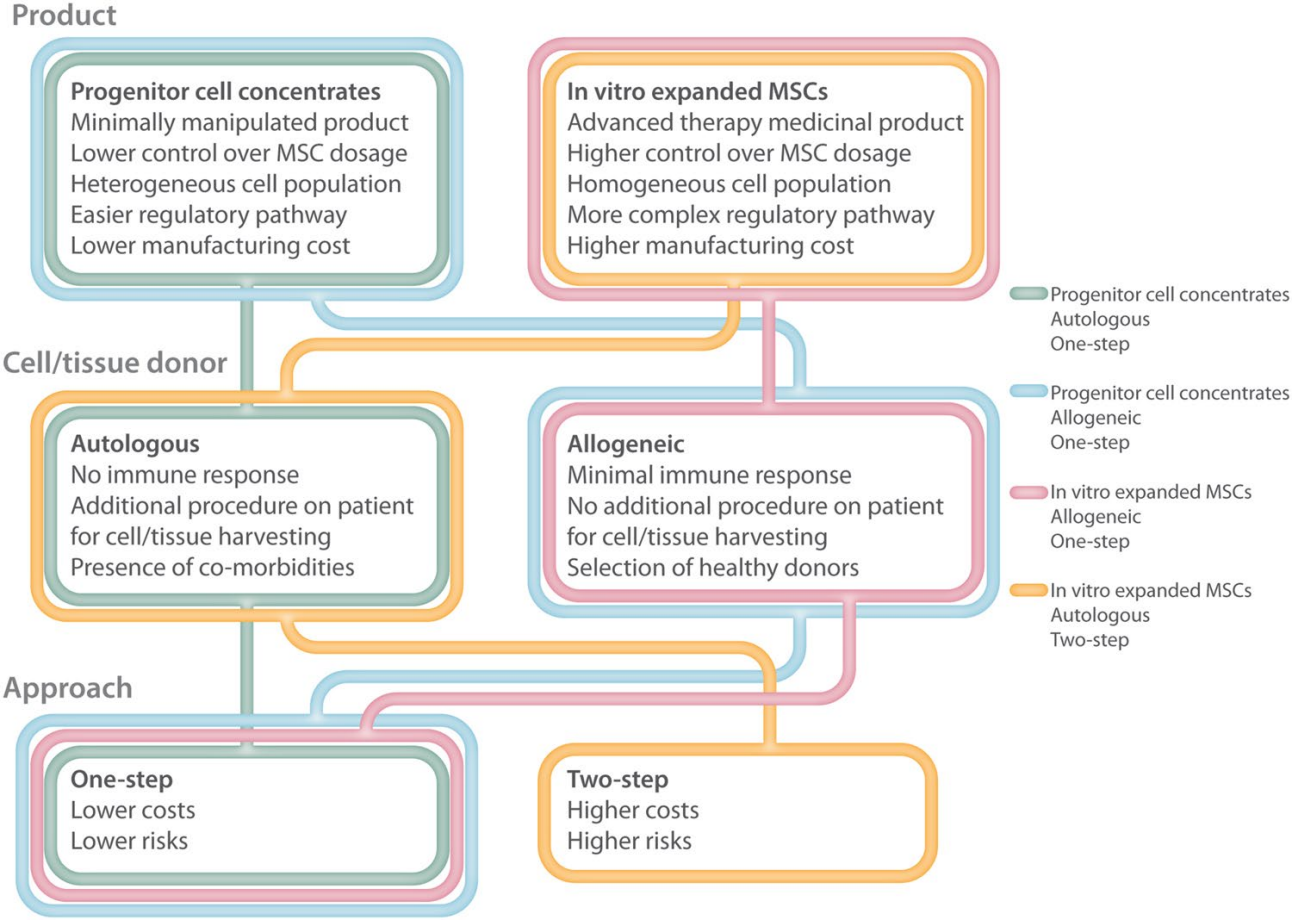
Treatment pathways of MSC-based injective treatments for OA: features of products and donor sources. Progenitor cell concentrates can be considered as one-step approach. In fact, in the case of autologous progenitor cell concentrates (pink), cell/tissue harvesting and patient treatment are performed on the same moment. Allogeneic progenitor cell concentrates (blue), similar to allogeneic in vitro expanded MSCs (green), are “off-the-shelf” products, compatible with a one-step intervention on the patient. The use of autologous expanded MSCs (yellow) is the only treatment that involves a two-step approach, since the patient undergoes cell/tissue harvesting and treatment in two separated moments. Professional illustration by Matilde Bongio, Ph.D., GoArts—IRCCS Istituto Ortopedico Galeazzi

The features of the different MSC-based treatment pathways are illustrated in Fig. 1. Besides bone marrow and adipose tissue, other tissues have recently gained interest as a source of MSCs [6]. Considering an allogeneic use of these cells, non-expanded MSCs isolated from amniotic fluid and membrane have been used for the treatment of OA. Indeed, the use of allogeneic MSCs is possible given their low immunogenicity, which express low levels of MHC class I molecules and lack the expression of MHC class II and other co-stimulatory molecules. Furthermore, MSCs can inhibit the activity of several types of immune cells via cell–cell contact and paracrine signaling, which avoids immune responses in allogeneic recipients [12]. This approach offers some advantages in terms of clinical outcome over autologous MSCs in old patients or in patients affected by co-morbidities whose MSCs may have reduced regenerative and therapeutic potential [13–16]. Moreover, the use of potential off-the-shelf commercial preparations of allogeneic MSCs may reduce the overall cost of cell therapies, while maintaining an accurate quality control. Yet, it is reasonable to think that this approach might gain relevance in the treatment of OA once a more extensive characterization of the efficacy and safety of allogeneic MSCs will be available, as recently showed in two studies for the treatment of focal chondral lesions [17, 18].

### Aim of the review

The purpose of this review paper is to provide the reader with the tools necessary to interpret the data, deriving from the available clinical studies concerning the intra-articular injection of MSCs, in the form of either expanded cells or progenitor cell concentrates, for the treatment of knee OA. We, indeed, believe that a good comprehension of the supposed mechanisms of action of MSCs might be very useful in the interpretation of the clinical effects of these treatments, as well in the critical analysis of the quality of the studies presented here. Reviewing the existing literature, we selectively focused on injective conservative procedures to evaluate the effects of the paracrine activity of MSCs in a pathological joint, aiming to answer the question: “Do the current results about the use of MSCs—either as freshly harvested or after culture expansion—for the treatment of OA support the supposed mechanisms of action of these cells?”. This analysis includes the papers, other than case reports, presenting patients affected by Kellgren–Lawrence (KL) grades I–IV, in which the injective treatment was never associated with surgical procedures of the affected knee(s). In this way, we tried to avoid confounding factors that may have made data interpretation even trickier. Tables 1, 2, 3, 4 provide the reader with an easy-to-consult summary of all the studies currently published in the literature following the aforementioned characteristics. Detailed information regarding the characteristics of the patient cohort, the study design, the assessments, and the main outcomes of each study is reported, while only the most relevant publications are commented in the following paragraphs.

**Table 1:** Summary of features and results of clinical studies applying expanded BMSCs for knee OA treatment

References	Cell donor	Patients			Study design				Study results		
		Number of patients	Knee OA grading	Age range	Type of study (ClinicalTrials.gov Identifier if applies)	Experimental group(s)	Cell dosage	Treatment comparator	Final follow-up	Measurements	Main outcomes
Davatchi [19]	Autologous	4	KL II–III	54–65 years	Phase I single-arm, open label (NCT00550524)	BMSC injection	8–9 × 10^6^ BMSCs	–	12 months	VAS Knee motion range Algofunctional assessments Patellae crepitus X-ray	Treatment safety ↓ Pain ↑ Knee function *Note: no statistical analysis on average data available*
Emadedin [20]	Autologous	6	KL IV	40–64 years	Case series	BMSC injection	20–24 × 10^6^ BMSCs	–	12 months	VAS WOMAC OA Index Knee flection Walking distance MRI	Treatment safety ↓ Pain ↑ Knee function ↑ Walking distance ↑ Cartilage thickness
Orozco [21]	Autologous	12	KL II–IV	29–75 years	Open-label, single-arm clinical trial (NCT01183728)	BMSC injection	40 × 10^6^ BMSCs	–	12 months	VAS WOMAC OA Index Lequesne Functional Index SF-36 quality-of-life questionnaire MRI	↓ Pain ↑ Knee function ↑ Cartilage quality
Orozco [22]	Autologous	12	KL II–IV	29–75 years	Phase I/II single-arm, open-label clinical trial (NCT01183728)	BMSC injection	40 × 10^6^ BMSCs	–	24 months	VAS WOMAC OA index Lequesne functional index SF-36 quality-of-life questionnaire MRI	Maintenance of the improvements achieved at 12 months and reported in [Orozco 2013]
Emadedin [23]	Autologous	6	KL III–IV	18–65 years	Case series	BMSC injection	0.5 × 10^6^ BMSCs/kg	–	30 months	VAS WOMAC OA index Walking distance MRI	Long-term safety ↓ Pain ↑knee function ↑ Walking distance ↑Cartilage quality
Vega [24]	Allogeneic (from single donors)	30 (15/treatment 15/control)	KL II–IV	36–73 years	Phase I/II multicenter, prospective, randomized, double-blind, comparator-controlled clinical trial (NCT01586312)	HA combined with BMSCs	40 × 10^6^ BMSCs	HA injection (control)	12 months	VAS WOMAC OA index Lequesne functional index SF-12 quality-of-life questionnaire MRI	In the experimental group (HA + BMSCs) ↓ Pain ↑ Knee function ↑ Cartilage quality No significant changes in the control group (HA)
Soler [25]	Autologous	15	KL II–III	33–63 years	Phase I/II prospective, open-label, single-dose, single-arm clinical trial (NCT01183728)	BMSC injection	40 × 10^6^ BMSCs	–	12 months Yearly VAS assessment for 4 years	VAS WOMAC OA index Lequesne functional index SF-36 quality-of-life questionnaire MRI	↓ Pain ↑ Knee function *Note: further reduction of VAS value at 4 year follow-up*
Davatchi [26]	Autologous	4	KL II–III	54–65 years	Phase I single-arm, open label (NCT00550524)	BMSC injection	8–9 × 10^6^ BMSCs	–	60 months	VAS Knee motion range Algofunctional assessments Patellae crepitus X-ray	Progressive loss of some of the improvements measured at 12 months [Davatchi 2011] *Note: no statistical analysis on average data available*
Lamo-Espinosa [27]	Autologous	30 (10/group 10/control)	KL II–IV	50–80 years	Phase I/II multicenter, randomized, comparator-controlled, open-label clinical trial (NCT02123368)	HA combined with two different BMSC doses	10 × 10^6^ BMSCs 100 × 10^6^ BMSCs	HA injection (control)	12 months	VAS WOMAC OA index Knee motion range MRI X-ray	In both HA + BMSCs groups ↓ Pain Only in HA + BMSCs (high dose) ↑ Knee function ↑ Cartilage quality No significant changes at 12 months in the control group (HA)
Gupta [28]	Allogeneic (pooled from multiple donors)	60 (10/group 20 placebo)	KL II–III	40–70 years	Phase II multicenter randomized, placebo-controlled, double-blind, clinical trial (NCT01453738)	HA combined with four different BMSC doses	25 × 10^6^ BMSCs 50 × 10^6^ BMSCs 75 × 10^6^ BMSCs 150 × 10^6^ BMSCs	Placebo	12 months	VAS WOMAC OA index Intermittent and constant OA pain MRI	Adverse events predominant for relevant BMSC doses (≥ 50 × 10^6^) Despite some improvements in the low-dose group, no significant changes compared to baseline or placebo
Al-Najar [29]	Autologous	13	KL II–III	34–63	Phase I prospective, open-label, clinical trial (NCT02118519)	Two BMSC doses injected 1-month apart from each other	1st dose: 30.8 × 10^6^ BMSCs 2nd dose: 30.4 × 10^6^ BMSCs	–	24 months	Knee injury and OA outcome score MRI	Treatment safety ↓ Pain ↑ Knee function Improvements in pain and knee function maintained from 6 to 24 months ↑ Cartilage thickness at 12 months

**Table 2:** Summary of features and results of clinical studies applying BMAC for knee OA treatment

References	Cell donor	Patients			Study design				Study results		
		Number of patients	Knee OA grading	Age range	Type of study (ClinicalTrials.gov Identifier if applies)	Experimental group(s)	Cell dosage	Treatment comparator	Follow-up	Measurements	Main outcomes
Kim [30]	Autologous	41 (75 knees)	KL I–IV	53–80 years	N/A	BMAC injected in combination with adipose tissue	N/A	–	12 months	VAS International knee documentation committee scale SF-36 quality-of-life questionnaire Knee injury and OA outcome score Lysholm score	↓ Pain ↑ Knee function Poorer outcomes in patients with KL IV compared to patients with OA at an earlier stage
Centeno [31]	Autologous	681 (840 knees)	KL I–IV	N/A Mean age reported for the two groups 54.3 years 59.9 years	Report based on registry data	BMAC injected in combination with PRP BMAC injected in combination with adipose graft and PRP	N/A	–	12 months	Numeric pain scale Lower extremity functional scale Improvement rating score	↓ Pain ↑ Knee function No difference between BMAC injection with and without adipose graft
Centeno [32]	Autologous	373 (424 knees)	KL I–IV	N/A Mean age reported for the two groups 54.5 years 50.2 years	Report based on registry data	BMAC with low cell content combined with PRP and platelet lysate BMAC with high cell content combined with PRP and platelet lysate	-> 4 × 10^8^ cells -≤ 4 × 10^8^ cells	–	12 months	Numeric pain scale Lower extremity functional scale International knee documentation committee scale Improvement rating score	↓ Pain ↑ Knee function Significantly higher pain reduction in patients treated with BMAC with high mononuclear cell content
Sampson [33]	Autologous	27 (bilateral knee OA) 46 (unilateral knee OA)	KL III–IV	23–79 years	Retrospective case series	BMAC injection followed by PRP injection (8 weeks later)	N/A	–	5 months	VAS Global patient satisfaction survey	↓ Pain High levels of patient satisfaction
Shapiro [34]	Autologous	25 (bilateral knee OA)	KL I–III	42–68 years	Prospective, randomized single-blind, placebo-controlled trial (NCT01931007)	Injection of BMAC combined with platelet-poor plasma	N/A	Placebo (contralateral knee)	6 months	VAS Intermittent and constant OA pain questionnaire	↓ Pain No significant difference in pain relief between knees treated with BMAC and with saline solution

**Table 3:** Summary of features and results of clinical studies applying expanded ASCs for knee OA treatment

References	Cell donor	Patients			Study design				Study results		
		Number of patients	Knee OA grading	Age range	Type of study (ClinicalTrials.gov Identifier if applies)	Experimental group(s)	Cell dosage	Treatment comparator	Final follow-up	Measurements	Main outcomes
Jo [35]	Autologous	Phase I: 9 (3/group) Phase II: 9	KL II–IV	18–75 years	Proof-of-concept clinical trial	Phase I: injection with three different ASC doses Phase II injection with the highest ASC dose	Phase I: 10 × 10^6^ ASCs 50 × 10^6^ ASCs 100 × 10^6^ ASCs Phase II: 100 × 10^6^ ASCs	–	6 months	Safety VAS WOMAC OA index Second-look arthroscopy Histology MRI	Treatment safe for all the tested ASC doses ↓ Pain and ↑ knee function only in the high-dose group
Pers [36]	Autologous	18 (6/group)	KL III–IV	50–75 years	Phase I multicentric, prospective, single-arm, open-label, dose escalating clinical trial (NCT01585857)	Injection with three different ASC doses	2 × 10^6^ ASCs 10 × 10^6^ ASCs 50 × 10^6^ ASCs	–	6 months	VAS WOMAC OA index Patient global assessment Knee injury and OA outcome score Short arthritis assessment scale SF-36 quality-of-life questionnaire	↓ Pain and ↑ knee function only in the low-dose group
Jo [37]	Autologous	Phase I: 9 (3/group) Phase II: 9	KL II–IV	18–75 years	2-year follow-up of the trial described in [35]	Phase I: injection with three different ASC doses Phase II injection with the highest ASC dose	Phase I: 10 × 10^6^ ASCs 50 × 10^6^ ASCs 100 × 10^6^ ASCs Phase II: 100 × 10^6^ ASCs	–	24 months	VAS WOMAC OA index Knee society clinical rating system Knee injury and OA outcome score MRI	At 1 year, significant improvements mainly in the high-dose group Only in the high-dose group, improvements maintained at 2 years
Song [38]	Autologous	Phase I: 18 (6/group) Phase II: 14 (the same patients treated in Phase I)	KL II–IV	40–70 years	Phase I/II randomized, double-blind clinical trial (NCT01809769)	Injection with three different ASC doses. Two injections at 3 and 6 weeks after liposuction A third ASC injection was provided after 48 weeks	Phase I: 10 × 10^6^ ASCs 20 × 10^6^ ASCs 50 × 10^6^ ASCs Phase II: third injection with 50 × 10^6^ ASCs	–	24 months	WOMAC OA index Numerical pain rating scale SF-36 quality-of-life questionnaire MRI	Treatment safe for all the tested ASC doses ↓ Pain, ↑ knee function, ↑ cartilage volume more relevant and long-lasting in the high-dose group The third injection increased the improvement rate, especially in patients previously treated with the low and middle ASC dose

**Table 4:** Summary of features and results of clinical studies applying adipose-derived SVF and microfragmented adipose tissue for the treatment of knee OA

References	Cell donor	Patients			Study design				Study results		
		Number of patients	Knee OA grading	Age range	Type of study (ClinicalTrials.gov Identifier if applies)	Experimental group(s)	Cell dosage	Treatment comparator	Final follow-up	Measurements	Main outcomes
Pak [39]	Autologous	2	N/A	70–79 years	Case series	Injection of SVF combined with PRP and dexamethasone, followed by weekly PRP injections for 1 month	N/A	–	3 months	VAS Knee motion range Functional rating index MRI	↓ Pain ↑ Knee function Note: no statistical analysis on average data available
Gibbs [40]	Autologous	4	N/A	23–50 years	Case series	Injection of SVF combined with PRP followed by monthly PRP injections for 4 months	11.5 × 10^6^ − 50 × 10^6^ cells	–	12 months	Knee injury and OA outcome score Physical function test	↓ Pain ↑ Knee function Note: no statistical analysis on average data available
Pak [41]	Autologous	3	KL III	60–87 years	Case series	Injection of SVF combined with HA + PRP followed by weekly HA + PRP injections for 3 weeks	N/A	–	5 months	VAS Knee motion range Functional rating index MRI	↓ Pain ↑ Knee function Note: no statistical analysis on average data available
Fodor [42]	Autologous	6 (8 knees)	KL I–III	51–69 years	Phase I open-label single-arm clinical trial (NCT02357485)	Injection of SVF	14.1 × 10^6^ cells	–	12 months	VAS WOMAC OA index Knee motion range MRI	↓ Pain ↑ Knee function Improvements at 3 months maintained at 12 months
Bansal [43]	Autologous	10 (13 knees)	KL I–II	≥ 50 years	Phase I open-label single-arm clinical trial (NCT03089762)	Injection of SVF combined with PRP	N/A	–	24 months	WOMAC OA index 6-min walk distance MRI	↓ Pain ↑ Knee function ↑ Walking distance Improvements maintained at 24 months
Hudetz [44]	Autologous	17 (32 knees)	KL II–IV	40–85 years	Prospective, open-label single-arm, clinical trial	Injection of micro-fragmented adipose tissue	N/A	–	12 months	VAS X-rays dGEMRIC MRI IgG glycome composition in blood plasma and synovial fluid	Treatment safety ↓ Pain ↑ Knee function ↑ Glycosaminoglycan content in residual areas of cartilage

## Why should MSCs work in OA?

The convenient but debated term “MSCs” has been used to describe virtually any ex vivo expanded stromal cell population. For more than 3 decades, the rationale for the use of MSCs in musculoskeletal applications has been their ability to differentiate into tissue-specific cell types, such as osteoblast-, chondrocyte-, and tenocyte-like cells. A dramatic cultural revolution started about 10 years ago when the scientists consistently focused their attention on the ability of these cells to “sense” the environment and secrete as a response large quantities of different bioactive molecules, such as cytokines, antioxidant and pro-angiogenic substances, trophic factors, and other proteins [45]. In physiological conditions, MSCs reside in the perivascular niche in a quiescent condition until the signals released after an injury activate their migration to the damaged site promoting the production of bioactive molecules to re-establish tissue homeostasis [46–48]. For this reason, they have been recently renamed as “medicinal signaling cells” [46, 47, 49]. As a result, the paracrine activity of MSCs would be involved in productive repair, by switching off inflammation, limiting stress response, and apoptosis, and recruiting the immune and reparative cells of the recipient [50–53]. An extensive in vitro and ex vivo research activity focused on the identification and explanation of the mechanisms of action of MSCs. Some of these studies clearly report an influence of MSC paracrine activity on inflammation and matrix turnover in OA, where the presence of a pro-inflammatory milieu was suggested as the switcher to promote the anti-inflammatory effects of MSCs. Indeed, priming BMSCs with OA synovial fluid promotes an increase of indoleamine 2,3-dioxygenase (IDO) expression [54], while priming these cells with IFNγ and TNFα determines an increase of IDO activity and IL-6 expression [55]. Moreover, conditioned medium obtained from BMSCs primed with OA synovial fluid inhibits T-cell proliferation [54], while, after IFNγ and TNFα priming, BMSC-conditioned medium determines IL-1β downregulation and SOCS1 (suppressor of cytokine signaling) upregulation in synovium explants, and a downregulation of ADAMTS5 and upregulation of IL-1Ra and SOCS1 in cartilage explants [55]. In addition, in the presence of high levels of pro-inflammatory mediators, a co-culture in transwell of ASCs obtained from infrapatellar fat pad, subcutaneous hip, or abdominal fat with chondrocytes and synoviocytes determines a decrease of IL-1β, IL-6, and CXCL8/IL-8 expression and release [56]. Finally, amniotic stem cells in co-culture with explants of cartilage and synovium have been shown to improve chondrocyte viability and cartilage glycosaminoglycan content as well as to provoke a shift of synovial macrophages towards an anti-inflammatory phenotype [57].

All these recent observations do not invalidate the “old school” theory based on the participation of MSCs to the repair process through their direct differentiation into tissue-specific cells. However, it is hard to believe that the few MSCs contained in a BMAC or SVF preparation injected intra-articularly can reach the multiple chondral lesions, permanently adhere and start the repair process by producing new cartilage. Therefore, while these two mechanisms of action of MSCs (paracrine action and direct cell differentiation) are not exclusive of each other, the difference is essential and needs to be taken into account for a critical analysis of the literature and for informing correctly the patients about the reasonable results they should expect from this kind of treatment.

## Clinical outcomes of MSCs in the treatment of OA

### Bone marrow-derived products

#### Bone marrow-derived MSCs (BMSCs)

Autologous expanded BMSCs have been the election choice in the majority of the clinical studies reported so far, although, on two small cohort of patients (*n* = 6), two studies of the same authors [20, 23] showed that the treatment with expanded BMSCs (20–24 × 10^6^ cells and 5 × 10^5^ MSCs/kg, respectively) allowed for an increase in cartilage thickness and extension of the repair tissue over the subchondral bone, as well as a significant improvement in Western Ontario and McMaster Universities OA Index (WOMAC) up to 30 months. These improvements remained stable up to 12 months with a following decrease, thus suggesting that subsequent injections of MSCs may be needed to achieve prolonged therapeutic efficacy. On the other hand, other studies have reported more durable outcomes of the intra-articular delivery of similar single doses of autologous expanded BMSCs. The injection of 40 × 10^6^ BMSCs in 12 patients with KL grades II–IV allowed for significant improvements over time of pain functional scores (VAS) and articular cartilage quality, without any decrease between 12- [21] and 24-month follow-up [22]. Another group reported the results up to 5 years [19, 26] post 8–9 × 10^6^ autologous BMSC injection on four patients with bilateral middle or advanced knee OA. Although a progressive deterioration was observed, at the last follow-up, the outcomes were still better than the baseline, thus suggesting a protective role of MSCs, since the untreated knee continued its progression towards degeneration. However, despite the advantage of having results at a very long follow-up for the same patients, due to the very limited number of patients and the lack of mean values and statistical analysis, it is impossible to draw any robust conclusion about the length of the therapeutic efficacy of the described procedure. A very recent study [29] has reported significant and stable improvements in terms of pain, knee function, and quality of life up to 24 months after two subsequent injections, with an interval of 1 month, of about 30 × 10^6^ BMSCs. Given the absence of contraindications in repeating this treatment, repeated doses of cells might be a solution to prolong the effectiveness of the results.

As can be inferred by the aforementioned studies and the others reported in Table 1, there is not a consensus about the ideal therapeutic dose for intra-articular treatment of OA. In the attempt to clarify this point, a phase I/II multicenter randomized-controlled trial at 12-month follow-up tested different doses of autologous expanded BMSCs (10 × 10^6^ and 100 × 10^6^) in association with hyaluronic acid (HA) in 30 patients with knee OA. Patients treated only with HA represented the control group [27]. Both doses of BMSC allowed for a significant VAS improvement with respect to baseline, where improvement in WOMAC was reported only for the patients treated with the highest dose. HA alone failed to improve symptoms at 12 months. Moreover, only the administration of the high dose of BMSCs halted the progressive loss of articular cartilage, indicating that a low dose of BMSCs may not be sufficient to obtain stable functional improvements and to significantly impact tissue quality.

The use of allogeneic BMSCs represents an alternative to autologous cell-based therapies. A recent randomized-controlled trial [24] showed significantly better results in 15 patients KL II–IV treated with intra-articular injection of 40 × 10^6^ allogeneic BMSCs in terms of VAS, WOMAC, Lequesne indices, and articular cartilage quality compared to the control group injected with HA only at 12-month follow-up. In a randomized double-blind multicentric placebo-controlled phase II study [28], four different doses of allogeneic BMSCs pooled from multiple donors (Stempeucel®) were tested on 10 patients KL II–III each, for a total of 40 patients, whereas the remaining 20 patients received a placebo injection. The lowest doses of BMSCs (25 × 10^6^ and 50 × 10^6^ cells) were safe and tolerated, while the highest dose groups (75 × 10^6^ and 150 × 10^6^ cells) yielded adverse events, mostly knee pain and swelling. Despite some positive trends in the 25 × 10^6^ group, none of the clinical parameters was significantly improved and no relevant changes in X-ray and MRI were observed compared to baseline. This indicates once again that the clinical efficacy of such therapies should be verified on a large patient cohort to achieve consistent results. Moreover, although MSCs are considered poorly immunogenic, still, they can elicit an immune response when used in allogeneic way, as shown by these results, and thus, the identification of a correct dose is even more crucial in this context.

#### Bone marrow aspirate concentrate (BMAC)

There are several commercial systems that allow clinicians to quickly recover concentrated, patient-derived nucleated cells, platelets, and other soluble factors in the form of BMAC. Most of these automated systems are based on gradient separation by centrifugation in a semi-closed or closed apparatus, and allow to achieve volume reduction and a 2–8X total nucleated cells with respect to the unprocessed bone marrow.

A recent single-blind placebo-controlled trial including 25 patients [34] showed a significant pain reduction after 6 months, but without difference with respect to placebo-treated contralateral knee. This lack of difference raises some concerns about the extent of the placebo effect when patients are included in a trial, but, at the same time, it raises several interpretative doubts; in fact, the pain relief at the contralateral (control) knee may have been affected by the reduction of symptoms on the target one, and also, since the same patient represented both the treatment and the control group, this may have led to a tricky subjective evaluation. Certainly, an objective evaluation of the patients might have helped to give a more accurate interpretation of the findings of this study. A retrospective case series including 73 patients with knee OA [33] with a 5-month follow-up showed that intra-articular injection of BMAC followed by PRP injection after 8 weeks resulted in a significant pain reduction and high patient satisfaction. However, cartilage quality was not assessed, and the combination of BMAC and the subsequent treatment with PRP does not allow distinguishing the therapeutic effect of BMAC alone. The data at 12-month follow-up of a registry including 373 (424 OA knees) patients that received BMAC injections for the treatment of OA showed significant improvements for all the reported pain and functional parameters compared to the baseline [32]. The authors set a threshold of 4 × 10^8^ cells to divide the patients in groups receiving a low dose and a high dose of total mononuclear cells, demonstrating more benefits in the high-dose group. This result seems to indicate that the number of progenitor cells, even when used without any cell expansion, could affect the outcomes. For this reason, the collection of data regarding mononuclear cell count in BMAC and an improvement in the standardization of cell counting would contribute to generate comparable data regarding the efficacy of BMAC injective treatments. It needs to be highlighted, indeed, that given the intraoperative setting of the use of BMAC, most of the studies about this approach do not provide any information about cell dose.

Besides cell content, also the stage of the disease appears to be a determinant in the outcome of these therapies. In a study conducted on 41 patients (75 knees) treated with BMAC injections in combination with adipose tissue used as a sort of scaffold to deliver more efficiently BMAC, the pain and functional scores improved in all the patients. Interestingly, the treatment yielded poorer results in patients with late-stage OA (KL IV) than in patients with the early/middle-stage OA (KL I–III) [30]. The combination of BMAC with adipose tissue was also analyzed in a registry reporting data of patients who underwent BMAC procedures with (224 procedures) and without (616 procedures) an adipose graft. While the pre- and post-treatment improvements were statistically significant in both groups, the differences between the groups were not, suggesting that addition of an adipose graft to BMAC did not provide any relevant benefit [31]. Again, this study only provides data relative to subjective algofunctional assessments, while it would be interesting to investigate if the addition of the adipose graft provides any improvement in articular cartilage quality to evaluate the risk–benefit ratio of performing an additional liposuction procedure.

### Adipose tissue-derived products

#### Adipose tissue-derived MSCs (ASCs)

The safety of high stem cell dosages for intra-articular injection has been investigated also in the context of adipose stem cell-based therapies. After having assessed the safety of three different autologous expanded ASCs' doses (10 × 10^6^, 50 × 10^6^, and 100 × 10^6^ cells) injected intra-articularly in patients affected by knee OA [35], a phase II study including nine additional patients treated with the highest cell dose was carried on. The results showed significantly better clinical results in the high-dose group with respect to the lower ones, suggesting that an adequate number of MSCs are crucial to achieve relevant clinical benefits. This result was further confirmed by a subsequent follow-up study, which reported that significant improvements at 2 years were maintained only in the high-dose group [37]. Completely opposite results were reported by another recent study [36] that tested different doses of autologous ASCs in a phase I clinical trial including 18 patients with symptomatic and severe knee OA. Of the three different ASC doses (2 × 10^6^, 10 × 10^6^, and 50 × 10^6^ cells), while all showed a satisfactory safety profile, significant improvements in terms of pain, function, and mobility were observed only in patients treated with the lowest dose of ASCs at 6-month follow-up. The apparent conflicting result of these studies [35, 36] may have been affected by a common bias, since the patients who exhibited the best response to ASC treatment had the worse baseline scores. Certainly, this may be ascribed to the lower expectations and a better predisposition to perceive any post-therapy improvements in these patients. However, these results can also be read as the need of an inflamed milieu to prime the injected ASCs and make them exert their homeostatic function at best, as demonstrated by ex vivo experiments. Once again, the heterogeneity and the limited number of patients included in the clinical trials preclude the possibility of a straightforward result interpretation, indicating that prospective trials on larger patients cohorts with a careful randomization based on the disease stage are needed. As already reported for BMSCs, repeated injections of 50 × 10^6^ ASCs allowed for significant improvements in terms of pain and knee function. An additional injection provided at 48 weeks after the previous ones was able to generate another improvement, positively affecting also the cartilage volume [38].

#### Stromal vascular fraction (SVF) and micro/nano-fragmented adipose tissue

A number of different systems to recover the “regenerative component” of adipose tissue have been recently introduced into the market. To comply with the rules of minimal manipulation, the tissue processing must avoid the use of enzymes or other molecules, and thus, generally, the digestion is mechanical. The isolation of SVF first requires a digestion of the extracellular matrix, usually followed by a centrifugation phase where SVF cells are concentrated. An alternative to the isolation of SVF is represented by the so-called micro- or nano-fragmented adipose tissue, belonging to the family of fat transfer. In this case, the extracellular matrix of the tissue is not removed, maintaining the tissue microarchitecture and an intact stem cell niche [58, 59].

Regarding the injection of SVF for the treatment of knee OA, a few studies showed improvements in the clinical and functional outcomes [39–41] up to 2-year follow-up, in some cases, also associated with radiological improvements. However, the very poor number of patients included in these studies and the concomitant use of other therapeutic agents like PRP, dexamethasone, or cavitation, do not allow for a clear identification of the effect of SVF. More recently, a study involving ten knee OA patients treated with SVF and PRP showed a reduction of pain, a functional improvement at 2 years of follow-up, and an increase of cartilage thickness after 1 year in six out of ten patients [43]. However, the quality of cartilage remains one of the main elements of discussion, given that, so far, just in a few cases, a stable hyaline cartilage was found after cartilage procedures. Therefore, it would be more appropriate to refer to cartilage repair. Positive effects have been reported also when using SVF alone. These include a functional improvement at 3 months and pain relief after 1 year shown in a study including 6 patients with knee OA [42]. Finally, a very recent study has reported the use of micro-fragmented adipose tissue in 17 patients (32 treated knees) showing significant improvements in terms of pain and cartilage quality up to 12 months [44]. Interestingly, this study used the dGEMRIC (delayed gadolinium-enhanced MRI of cartilage) protocol for the MRI assessment to determine changes pre- and post-treatment in glycosaminoglycan content in specific cartilage regions, as a measure of the trophic and paracrine actions of progenitor cells on resident chondrocytes, which yields more specific information than cartilage thickness assessed with standard MRI. Despite these encouraging evidences, the relatively low number of patients enrolled in these studies does not allow withdrawing definitive conclusions about treatment efficacy and further studies on larger patient cohorts are required to select the best strategy/device to use  and demonstrate the long-term efficacy of this approach.

### Alternative source of MSCs

#### Amniotic fluid cells

Amniotic suspension allografts (ASAs), containing particulate human amnion and amniotic fluid cells, have also been proposed for the treatment of symptomatic knee OA [60]. In an open-label prospective study on six patients with knee OA, it was demonstrated that a single intra-articular ASA injection from allogeneic donors allowed for significant improvements in pain and functional scales observed up to 12 months. These results indicated this novel cell source as an alternative tool for OA treatment, paving the way to a larger, placebo-controlled randomized trial to further assess treatment efficacy that recently completed patients’ enrolment.

## The lesson learned

### What we should expect from OA patients treated with MSCs?

A not negligible number of studies assessing the efficacy of MSC, either freshly harvested or culture-expanded, for the treatment of OA have been published so far. This demonstrates the interest of the scientific community for this conservative approach that may potentially change the treatment scenario of this very common and disabling disease. Considering the in vitro and in vivo findings reported so far, when treating an OA joint, we should expect a resolution of symptoms, at least transient, given the ability of MSCs to sense the environment and secrete, accordingly, a plethora of trophic and immunomodulatory molecules. This has been, indeed, reported in most of the studies analyzed here, although with different extent and durability. Durability of this treatment and more in general of all the “biological” treatments included PRP is one of the main points of discussion. The detractors of MSC-based treatments criticize the duration of the effects, that in some cases was less than a year, whereas in other cases lasted up to 2 years [22, 37, 43], with preliminary evidences of even a longer effect although progressively decreasing with time [26]. However, it should not be surprising that these treatments cannot lead to a definitive resolution of the disease. Indeed, injecting these therapeutic agents locally, it is just possible to modulate the microenvironment the cells found once delivered in the joint, without being able to counteract the inexorable progression of OA. MSCs, differently from PRP that has a short in vivo half-life [61], can survive longer in a joint cavity and keep releasing molecules. However, it is hard to predict the length of MSCs life once delivered, given the hostile microenvironment which they encounter, which is often hypoxic, rich of inflammatory mediators, and, sometimes, has a low pH, being thus characterized by sub-optimal conditions for MSC survival [62]. This, together with many other reasons amongst which the actual number of cells delivered, the severity of the disease, and the general joint/patient condition, can be a partial explanation of the different duration of the results reported in the literature. In any case, improving the patients’ quality of life for at least 1 year, but very often even for a longer period, should be consider a satisfactory result, especially for those patients who have been suffering from symptoms for long time and were used to assume daily analgesic and/or anti-inflammatory drugs with the well-known side effects. Moreover, given the absence of contraindication in repeating these treatments for a number of times, the use of repeated cell injections upon symptoms which return appears a reasonable approach to take full advantage of this technique.

It is well known that there is a high subjectivity of the patient response to the conventional synthetic drugs, and this is strongly dependent on the patient’s characteristics, since the drug formulation is consistent and standardized. When receiving a MSC treatment, the grade of complexity in predicting the patient’s response dramatically increases given the lack of standardization of the MSC preparation, especially those prepared at the point of care, which may affect the features of the final product. This does not mean that MSCs do not have therapeutic effects. Indeed, in our opinion, the lack of result homogeneity highlighted by some review papers [63] may be expected and not necessarily intended as a negative finding. For this reason, we do not agree with the authors that expect to find unambiguous proof of MSC efficacy comparing different types of studies involving dramatically different patient types and sometimes comparing the therapeutic outcome in different joints. Rather, we believe in a correct interpretation of the findings of the single studies that may lead to important conclusions if they are well designed. Moreover, likewise PRP, the quality and properties of BMC and SVF/micro-fragmented adipose tissue are strongly affected by the method of preparation and device used. For this reason, once again, it would be methodologically uncorrected to pool the results of different techniques, which, instead, need to be analyzed separately.

While the medical community well accepts the idea of chronic pharmacological protocols to give a stable relief from the target disease, it seems harder to accept the idea that the effects of a one-shot cell-based treatment cannot last forever. What we should have learned so far is that we cannot aspire to compare cell-based products, above all freshly prepared cell concentrates, with the conventional drugs, given the aforementioned substantial differences. At the same time, interpreting the results of cell-based treatments cannot even be compared to surgical treatments such as joint replacement, that, of course, provide much more durable results but imply an incomparable invasiveness and possible side effects which many patients are not ready or not yet in the need to face.

### How could we improve the knowledge about MSCs’ treatment for knee OA?

**Fig. 2:**
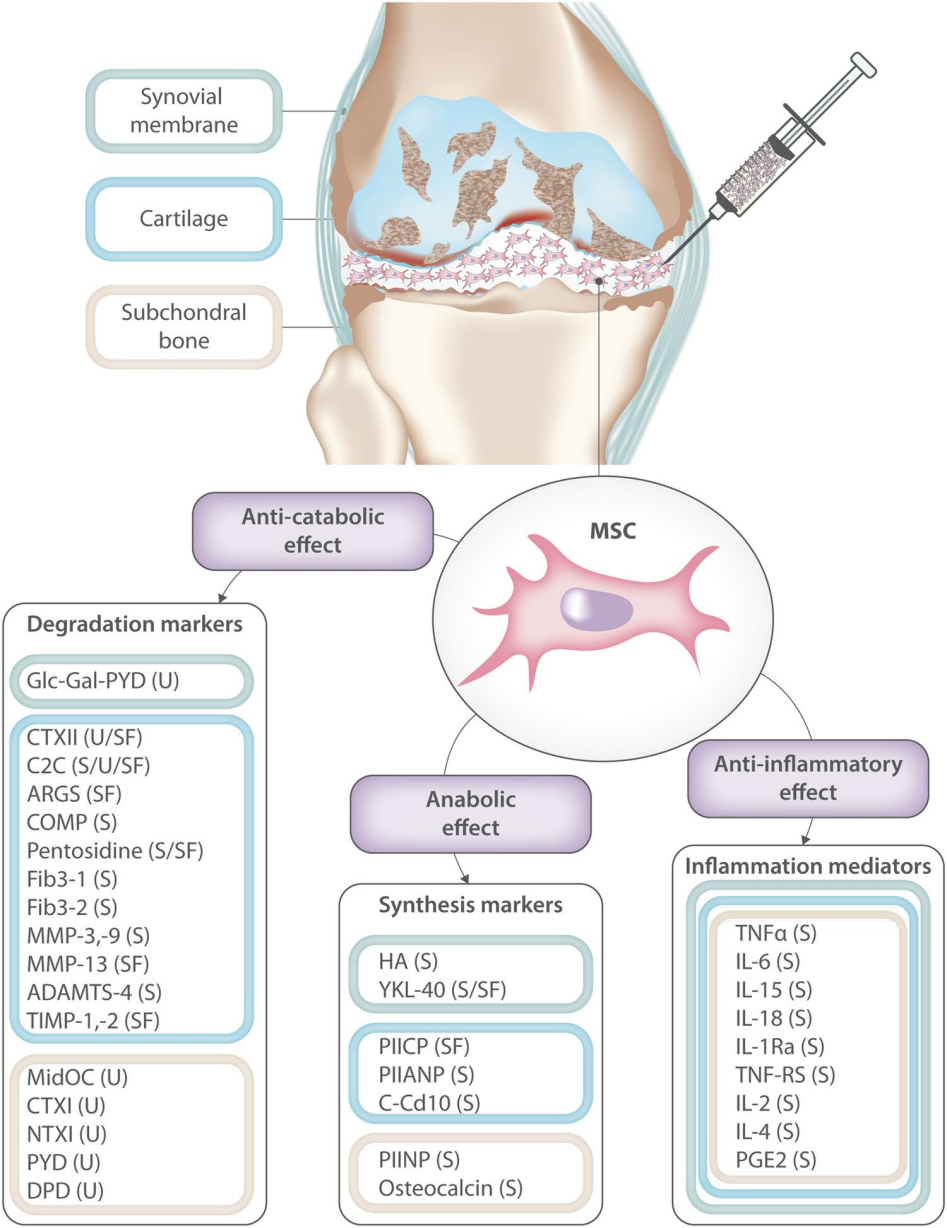
Synovial, cartilaginous, and bony-derived markers of degradation, synthesis, and inflammation in a joint affected by OA [64]. The paracrine activity of the MSCs in the OA articular environment resides in their anti-inflammatory, anti-catabolic, and trophic abilities. Monitoring of variations in these markers has been proposed as a strategy to evaluate the efficacy of MSC-based OA treatments. Professional illustration by Matilde Bongio, Ph.D., GoArts— IRCCS Istituto Ortopedico Galeazzi

While we acknowledge that the inconsistency of the results reported in the literature is affected by the intrinsic characteristics of this treatment, at the same time, we claim for a more rigorous approach in conducting studies. Among all the studies reported (Tables 1, 2, 3, 4), only four of them (14%) present a control group such as HA or placebo. Even worse, more than one-third (37%) of the studies show the results on less than ten patients, and thus, considering the high inter-patient variability hardly allows for any deduction. More controlled trials as well as multicentric studies allowing to recruit higher number of patients are needed, especially for adipose-derived MSC treatments, which have been more recently introduced with respect to bone marrow ones. The design of these studies is crucial and deserves much attention to achieve consistent and easy-to-interpret outcomes. The satisfactory, somehow striking results, obtained by scientists in vitro and in vivo encouraged the rapid translation of MSC-based therapies. However, while it is easy to provide an optimal in vitro setting for MSCs to grow and perform, as well as to analyze the objective results in animal models (histological/biochemical analysis), the clinical setting is far from offering these possibilities, and thus, translating these approaches into successful clinical protocols has proven to be trickier than probably expected. This line of investigation is particularly challenging in the absence of tools that allow the identification of objective improvements following MSC treatments. Hence, the scientific community urgently needs to define a panel of standard outcome measures and suitable time points to evaluate the efficacy of the therapy. Specific MRI protocols, such as dGEMRIC, have been proven to be more sensitive and give more conclusive information about the actual quality of the repaired cartilage, instead of only focusing on cartilage thickness, which, not surprisingly, may not be affected by this treatment, especially in older patients, or affected by severe OA. Rather, new and more specific tools should be used to evaluate the possible modulation of the local environment after a MSC treatment. Amongst them, biomarkers that can be assessed in body fluids, such as blood plasma, urine, and synovial fluid (Fig. 2), represent an invaluable and non-invasive tool to monitor over time the efficacy of MSC-based treatments. Monitoring the variations in biomarkers will allow to specifically focus on the trophic and/or immunomodulatory activity of MSCs, thus shading further light on the in vivo mechanisms of action of these biological therapeutics and correlating them with the observed clinical improvements. To note that accurate longitudinal studies might be very useful too, as they would provide data on a large variety of patients, which later can be stratified and analyzed. In this view, the creation of common registries shared by a group of centers and including relevant patients data and information that would allow for a critical outcome interpretation seems to be a smart option to further improve the knowledge in this field.

## Conclusions

Although substantial data have been published to date mostly accompanied by satisfactory results, the complexity of MSC metabolism and related therapeutic effects does not allow withdrawing definitive conclusions about the superiority of one tissue source over another, as well as about the best cell dose (if measurable) and the long-term durability of the effects of these procedures. Despite this, we are convinced that MSCs will have an important role in the conservative treatment of OA and that the research needs to continue to improve our knowledge. From a practical point of view, although less explored, one-step procedure, implying the use of autologous unexpanded sources of MSCs, presents advantages that cannot be questioned in comparison with two-step approaches. Much work needs to be done to carefully define the clinical circumstances of OA joints, but even more the characteristics of patients where these techniques should be utilized as well as their in vivo mechanisms of action. This should be achieved using adjunctive innovative technologies, such as cellular and molecular approaches, to define a complete picture of the patients in terms of local and/or systemic levels of inflammatory and metabolic markers of pathology. A more accurate monitoring of the complexity of the OA biology will further help in the early diagnosis and in the evaluation of its evolution. To accomplish this task, a strict collaboration between basic scientists, clinicians, industry, and regulatory agencies is needed to gain a better understanding of the very complex phenomena behind MSC therapeutic effects.

With a look towards the next future, the assumption that the therapeutic effect of MSCs in the treatment of OA relies on their secreting response to the local microenvironment signals clears the way to isolate the MSC-derived “healing” factors, the so-called secretome. The use of secretome would mean to take advantage of the product secreted by the cells without the use of the whole living cells, allowing to avoid the risks and discomfort of cellular transplantation. While the secretome will permit overcoming some of the limitations of cell-based therapies, it will deserve even more attention before being implemented in clinical setting given the need of an in vitro preparation and standardization. The current insights should help the scientific community to design more informative and revealing experiments that will lead to a more accurate translation into practical and effective clinical treatments. This field of investigation is very active and the promising preliminary evidences have been already observed in animal models of different diseases, confirming the feasibility of the approach that may demolish further barriers in the use of MSC-derived products.

## Abbreviations

OA: Osteoarthritis
MSC: Mesenchymal stem cell
BMSCs: Bone marrow-derived mesenchymal stem cell
ASCs: Adipose-derived mesenchymal stem cell
BMAC: Bone marrow concentrate
SVF: Stromal vascular fraction
MRI: Magnetic resonance imaging
VAS: Visual analog scale
WOMAC: Western Ontario and McMaster Universities osteoarthritis index
HA: Hyaluronic acid
PRP: Platelet-rich plasma
ASA: Amniotic suspension allograft
